# Accuracy of a reverse dot blot hybridization assay for simultaneous detection of the resistance of four anti-tuberculosis drugs in *Mycobacterium tuberculosis* isolated from China

**DOI:** 10.1186/s40249-020-00652-z

**Published:** 2020-04-16

**Authors:** Li Wan, Qian Guo, Jian-Hao Wei, Hai-Can Liu, Ma-Chao Li, Yi Jiang, Li-Li Zhao, Xiu-Qin Zhao, Zhi-Guang Liu, Kang-Lin Wan, Gui-Lian Li, Cha-Xiang Guan

**Affiliations:** 1 Department of Physiology, Xiangya School of Medicine, Central South University, Changsha, Hunan 410078 China; 2 State Key Laboratory for Infectious Disease Prevention and Control, Collaborative Innovation Center for Diagnosis and Treatment of Infectious Diseases, National Institute for Communicable Disease Control and Prevention, Chinese Center for Disease Control and Prevention, Beijing 102206, People’s Republic of China; 3 Department of Molecular Biology, Shanghai Jiaotong University Affiliated Sixth People’s Hospital, Shanghai 200233, People’s Republic of China; 4 Department of Clinical Laboratory, Shanghai Public Health Clinical Center, Fudan University, Shanghai 201508, China

**Keywords:** *Mycobacterium tuberculosis*, Drug resistance, Reverse dot blot hybridization, Isoniazid, Rifampicin, Streptomycin, Ethambutol

## Abstract

**Background:**

Drug resistant tuberculosis poses a great challenge for tuberculosis control worldwide. Timely determination of drug resistance and effective individual treatment are essential for blocking the transmission of drug resistant *Mycobacterium tuberculosis.* We aimed to establish and evaluate the accuracy of a reverse dot blot hybridization (RDBH) assay to simultaneously detect the resistance of four anti-tuberculosis drugs in *M. tuberculosis* isolated in China.

**Methods:**

In this study, we applied a RDBH assay to simultaneously detect the resistance of rifampicin (RIF), isoniazid (INH), streptomycin (SM) and ethambutol (EMB) in 320 clinical *M. tuberculosis* isolates and compared the results to that from phenotypic drug susceptibility testing (DST) and sequencing*.* The RDBH assay was designed to test up to 42 samples at a time. Pearson’s chi-square test was used to compute the statistical measures of the RDBH assay using the phenotypic DST or sequencing as the gold standard method, and Kappa identity test was used to determine the consistency between the RDBH assay and the phenotypic DST or sequencing.

**Results:**

The results showed that the concordances between phenotypic DST and RDBH assay were 95% for RIF, 92.8% for INH, 84.7% for SM, 77.2% for EMB and the concordances between sequencing and RDBH assay were 97.8% for RIF, 98.8% for INH, 99.1% for SM, 93.4% for EMB. Compared to the phenotypic DST results, the sensitivity and specificity of the RDBH assay for resistance detection were 92.4 and 98.5% for RIF, 90.3 and 97.3% for INH, 77.4 and 91.5% for SM, 61.4 and 85.7% for EMB, respectively; compared to sequencing, the sensitivity and specificity of the RDBH assay were 97.7 and 97.9% for RIF, 97.9 and 100.0% for INH, 97.8 and 100.0% for SM, 82.6 and 99.1% for EMB, respectively. The turnaround time of the RDBH assay was 7 h for testing 42 samples.

**Conclusions:**

Our data suggested that the RDBH assay could serve as a rapid and efficient method for testing the resistance of *M. tuberculosis* against RIF, INH, SM and EMB, enabling early administration of appropriate treatment regimens to the affected drug resistant tuberculosis patients.

## Background

*Mycobacterium tuberculosis* infection remains a global public health threat due to its high risk of transmission, morbidity and mortality [1]. Timely case identification and appropriate treatment is of great significance in blocking the transmission of *M. tuberculosis*, especially for the prevention of multidrug-resistant (MDR) or extensively drug-resistant (XDR) *M. tuberculosis* emergence. Generally, culture-based phenotypic drug susceptibility testing (DST) methods are currently the gold standard for drug resistance detection and reliable and reproducible for certain anti-tuberculosis medicines, but these methods are time-consuming, e.g., MGIT medium based DST needs 4–13 days and Lowenstein-Jensen (L-J) slants based DST needs 6 weeks [2]. Therefore, nucleic acid-based antibiotic susceptibility tests, which can be performed within 1 or 2 days, are increasingly considered as a diagnostic alternative.

Knowledge on the mutation profiles of drugs will be helpful to establish the molecular diagnosis assay for detecting the drugs resistance. Previous data shown that rifampicin (RIF) resistance is mainly attributed to the mutations within the RIF resistance-determining region (RRDR) of *rpoB* (encoding β-subunit of RNA polymerase) with codons 531, 526 and 516 being the most prevalent sites [3–5]. Mutations in this region account for more than 90% of RIF resistance [5]. The molecular mechanisms of isoniazid (INH) resistance involve several genes in multiple biosynthetic networks and pathways. Mutation in the *katG* gene is the major cause for INH resistance, followed by *inhA* promoter and *oxyR-ahpC* intergenic region [6]*.* A study analyzed on 1219 INH resistant isolates shown that the most frequent mutation loci were *katG* 315 (78.1%), promoter *inhA* (− 15) (22%) [7]. Mutations in *oxyR-ahpC* intergenic region were found attributed to 10–15% INH resistance [7–9]. Mutations in *rrs* and *rpsL* genes, which are involved in the synthesis of 16S rRNA and the ribosomal protein S12, respectively, have been shown to be responsible for 50–95% streptomycin (SM) resistant strains [4, 7, 8]. The most frequent loci in *rpsL* and *rrs* associated with SM resistance were *rpsL* 43, *rpsL* 88, *rrs* 513 loop and 912 loop [4, 7, 8]. Resistance to ethambutol (EMB) is mainly mediated by nucleotide changes in *embB* particularly in codons 306 and 406 which accounted for 38–73% EMB resistance [4, 7, 8]. These resistance-associated mutations provide the basis for molecular diagnostic approaches. In fact, the World Health Organization (WHO) recommends the use of molecular assays that target the specific mutations associated with resistance to certain anti-tuberculosis drugs [10, 11]. GenoType MTBDR*plus* (Hain Lifescience GmbH, Nehren, Germany), which is a commercially available line probe assay, can detect *M. tuberculosis* complex as well as predict resistance to RIF and INH simultaneously from one to 16 samples within 5–6 h, either in isolates or smear-positive specimens [11, 12]. Most recently, the FluoroType MTBDR (FluoroType) assay from Hain Lifescience GmbH is designed as a qualitative in vitro test for the automated detection of the *M. tuberculosis* complex and resistance to RIF and INH directly from sputum specimens [13]. Another new version (v2.0) of GenoType MTBDR*sl* assay (Hain Lifescience GmbH, Nehren, Germany) was developed to detect resistance to fluoroquinolones and second-line injectable drugs [14]. However, all of these assays did not include probes aimed for detecting mutations in *oxyR-ahpC*, which has been reported accounted for 10–15% INH resistance [7–9]. Besides, detection of SM resistance is not included in GenoType methods. Therefore, it is necessary to establish a new assay to detect more multiple genes and mutations in a timely manner.

The in-house reverse dot blot hybridization (RDBH) assay has been widely used in the spoligotyping technique for *M. tuberculosis* lineage identification. Similarly, this technique had been successfully applied for the detection of mutations related to resistance to RIF and shown a sensitivity of 91.2% and a specificity of 98.3% comparing to phenotypic DST in our lab [15]. In order to further simplify consumables and make the procedure easier to perform, we improve the imaging method by replacing the enhanced chemiluminescence detection system described in previously report [15] with TMB (3, 3′,5,5′-tetramethylbenzidine) reagent, which develop visible colorful spot directly. The RDBH method was further optimized to detect mutations conferring RIF, INH, EMB and SM resistance simultaneously by targeting in seven genes: *rpoB* (for resistance to RIF)*, katG, inhA* promoter and *oxyR-ahpC* (INH)*, rrs* and *rpsL* (SM) and *embB* (EMB), and evaluated its efficiency and accuracy to predict the resistance of four drugs by comparing to the phenotypic DST and sequencing results in 320 clinical *M. tuberculosis* isolates.

## Methods

### *Mycobacterium tuberculosis* strains

A total of 320 *M. tuberculosis* isolates were selected from the strain bank of the National Institute for Communicable Disease Control and Prevention, Chinese Center for Disease Control and Prevention. These isolates were obtained from 320 adult patients with pulmonary tuberculosis from 2005 to 2011 from institutes for tuberculosis control and prevention as well as tuberculosis hospitals distributed in six provincial-level administration divisions (PLADs) of China. The numbers isolated from each PLAD were with the following: Fujian, 76; Henan, 17; Hunan, 70; Inner Mongolia: 15; Sichuan, 34; Tibet, 108. H37Rv (ATCC 27294) was used as the reference strain.

All the strains were stored in physiological saline containing 50% glycerol at − 70 °C. Prior to characterizing the drug susceptibility, the strains were recovered on L-J medium for 4 weeks at 37 °C. The isolate profiles of drug susceptibility were reevaluated in our laboratory by the proportion method using L-J slants with the following: 0.2 μg/ml for INH, 40 μg/ml for RIF, 4 μg/ml for SM, and 2 μg/ml for EMB [16]. Of 320 isolates, 78 were susceptible to the four drugs, 23 were mono-INH resistant, eight were mono-SM resistant, 11 were mono-RIF resistant, 160 were MDR isolates (resistant to at least RIF and INH), 40 were poly-drug resistant (resistant to more than one drug but not MDR). In total, 206 were INH-resistant, 185 were RIF-resistant, 83 were EMB-resistant and 155 were SM-resistant.

### Genomic DNA extraction

*M. tuberculosis* genomic DNA was extracted from fresh cultures growing on L-J slants. The bacterial cells were harvested and transferred to microcentrifuge tubes containing 200 μl TE buffer (10 mmol/L Tris-HCl and 1 mmol/L EDTA, pH 8.0), then inactivated in a 95 °C water bath for 10 min and incubated at 85 °C for 30 min. After centrifugation for 5 min at 13 523×*g*, the supernatant containing DNA was collected and stored at − 20 °C for further use.

### Multiplex PCRs

The RDBH were designed based on multiplex PCRs. Seven PCR primer pairs (*rpoB*, *katG*, *inhA* promoter, *oxyR-ahpC*, *rpsL, rrs* and *embB*), biotinylated at the 5′ end were designed to work together in a multiplex reaction. The primer sequences and amplicon sizes were listed in Table 1. Amplifications were performed in a final volume of 50 μl containing 25 μl 2 × Hot Start *Taq* Master Mix (Sinobio, Shanghai, China), 2 μl DMSO, 10–100 ng of genomic DNA, forward and reverse primers with following concentraions (*rpoB*, 0.12 μmol/L; *katG*, 0.18 μmol/L; *inhA* promoter, 0.2 μmol/L; *oxyR-ahpC,* 0.2 μmol/L; *rpsL*, 0.3 μmol/L; *rrs*, 0.1 μmol/L; *embB*, 0.24 μmol/L). The cycling condition was as follows: 10 min at 94 °C, 35 cycles of 1 min at 94 °C, 1 min at 63 °C and 140 s at 72 °C, and 10 min at 72 °C. The PCR products were analyzed by electrophoresis using 3% agarose gels at 120 V for 80 min.

**Table 1:** Primers designed for multiplex PCRs and sequencing

Drug	Gene	Primer	Sequence (5′ → 3′)	Amplicon size (bp)
RIF	*rpoB*	*rpoB*-F	GGTCGCCGCGATCAAGGAGT	228
		*rpoB*-R	GAGCCGATCAGACCGATGTT	
INH	*katG*	*katG*-F	CAGATGGGCTTGGGCTGGAA	152
		*katG*-R	TTCGTCAGCTCCCACTCGTAGC	
	*inhA*	*inhA*-F	TGGTCGAAGTGTGCTGAGTC	193
		*inhA*-R	TCCGGTAACCAGGACTGAAC	
	*oxyR-ahpC*	*oxyR-ahpC*-F	GCAGTCACAACAAAGTCAGCTCTG	401
		*oxyR-ahpC*-R	ACAGGTCACCGCCGATGAGA	
SM	*rpsL*	*rpsL*-F	TTGTGGTTGCTCGTGCCTG	635
		*rpsL*-F	CAACTGCGATCCGTAGACCG	
	*rrs*	*rrs*-R	CTCTCGGATTGACGGTAGGTGG	540
		*rrs*-F	GCGTCCTGTGCATGTCAAACC	
EMB	*embB*	*embB*-F	CGTGGTGATATTCGGCTTCCTG	493
		*embB*-R	CTGCACACCCAGTGTGAATGCG	

*RIF* Rifampicin, *INH* Isoniazid, *SM* Streptomycin, *EMB* Ethambutol

### RDBH assay

By targeting the major gene mutation sites conferring drug resistance to four anti-tuberculosis drugs including INH, RIF, SM and EMB, 15 probes detecting wild type (WT) sequences and 18 probes detecting mutant (MT) sequences were newly designed by using Primer Premier v.5.0 (Premier Biosoft International, Palo Alto, USA). The lengths of probes were adjusted to guarantee the difference of the melting temperatures of all probes within 6 °C so that they could be processed under the same hybridization and washing conditions. All the probes were covalently bonded to the negatively charged nylon membrane (Biodyne C, Pall Corporation, USA). 30 μl of each PCR product was diluted in 140 μl 5 × saline-sodium phosphate-ethylenediaminetetraacetic acid (SSPE) /0.1% sodium dodecyl sulfate (SDS) buffer, heat-denatured at 100 °C for 10 min then immediately cooled on ice. The denatured single-stranded DNA was applied on the membrane in the miniblotter slots (Immunetics, Cambridge, MA, USA) and hybridized with probes at 60 °C for 1 h. The membrane was washed twice with 2 × SSPE/0.5% SDS buffer at 50 °C and subsequently incubated at 42 °C for 40 min with 20 ml 2 × SSPE/0.5% SDS containing 1:4000 diluted peroxidase (POD). Then the unbound conjugate was removed by washing twice in 2 × SSPE/0.5% SDS for 10 min at 42 °C, and rinsed once with 2 × SSPE for 5 min at room temperature. Finally the membrane was developed by incubating with 1 ml TMB (Beyotime, Shanghai, China) reagent for 5 min in the dark.

A clear visible blue-green spot was recorded as positive. Clinical isolate was considered to be susceptible to the drug when the WT probes reacted positively while the MT probes were negative. When the mutant probe had a stronger color than the corresponding WT probe, the strain was considered to be a mutant genotype and therefore resistant to the drug. A strain without positive spots of WT probes was recognized to have a specific mutation, and interpreted as resistant to the drug. Two H37Rv pan-susceptible samples were used as positive controls and water was employed as negative control. The assay was performed and read in a double-blind way. For the disagreement results between two readers, a third reader was included to make the final decision. The results from the RDBH assay were compared to that obtained by proportion method (phenotypic DST) and sequencing.

### Standardization and validation

H37Rv reference strain (ATCC 27294) and several *M. tuberculosis* clinical isolates with known mutations were used to select the optimal probe sequences and standardize the probe concentrations as well as assay conditions. Over a period of 2 years, series of experimental conditions according to the previous study of our lab, with varying probe concentrations (0.1–1 μmol/L), different hybridization temperatures (50–65 °C), washing temperatures (45–60 °C) and color-developing systems including alkaline phosphatase-nitroblue tetrazolium chloride/5-bromo-4-chloro-3-indolyl pholsphate (AP-NBT/BCIP) and POD-TMB were performed in pilot experiments. A total of 320 *M. tuberculosis* clinical isolates was used to assess the performance of the RDBH assay, and the results were compared to that obtained by phenotypic DST and sequencing methods.

### Sequencing

Mutations in seven genes or regions (*rpoB*, *katG*, *inhA* promoter, *oxyR-ahpC*, *rpsL, rrs* 513 loop and *embB*) were also determined by sequencing. The primer sequences and concentrations used for separate PCR were equal to that used in multiplex-PCRs with the exception of primers of *katG* for obtaining longer DNA sequence: forward- AATCGATGGGCTTCAAGACG, reverse- CTCGTAGCCGTACAGGATCTCG [17]. The PCR products of each gene were characterized by sequencing using the forward primers on an ABI Prism 3730 automated DNA sequencer (ABI Prism, Carlsbad, CA, USA). The resulting DNA sequences were analyzed using the basic local alignment search tool (http://www.ncbi.nih.gov/BLAST), and the specific mutations in protein sequences of the individual isolates were identified.

### Data analysis

The Pearson’s chi-square test was used to compute the statistical measures of sensitivity, specificity, positive predictive value (PPV), negative predictive value (NPV), and concordance of the RDBH assay using the phenotypic DST or sequencing as the gold standard method. The consistency analysis on the results of the different methods was conducted by Kappa identity test. The Kappa value was interpreted as follows: < 0.4, limited; 0.41–0.75, moderate; ≥ 0.75, excellent [18]. All statistical analyses were performed using SPSS 18.0 software (SPSS Inc., Chicago, IL, USA).

## Results

**Fig. 1:**
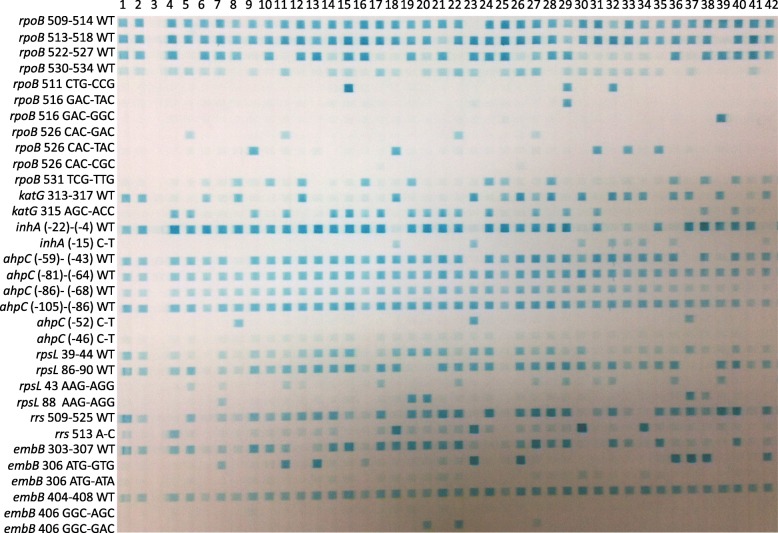
The hybridized image detected with the reverse dot blot hybridization assay. Note: lanes 1 to 2: H37Rv reference strain; lanes 3: Negative control; lanes 4 to 42: *Mycobacterium tuberculosis* clinical isolates. The detail information on how to interprete the results of the RDBH assay, the phenotypic resistance and sequencing results for 41 *M. tuberculosis* were shown in supplemental Table 1

At the present study, we built a RDBH assay based on multiplex PCRs and evaluated its accuracy comparing with phenotypic DST and sequencing. The RDBH assay can test up to 42 *M. tuberculosis* DNA samples at a time, the turnaround time from the beginning of multiplex PCRs to provide resistance results of isolates was 7 h. The results of agarose gel electrophoresis shown that each target fragment was successfully amplified. A total of 320 *M. tuberculosis* were accessed by the RDBH assay. The RDBH results were determined according to the blot on the hybrid membrane, the interpretation on INH susceptibility between two readers was identical, whilst disagreements were found on five, six and six out of 320 isolates on SM, EMB and RIF susceptibility interpretations, respectively, and the third reader was needed to determin the final results. Figure 1 showed the RDBH membrane results of 41 isolates.

The performance of the RDBH assay compared with phenotypic DST and sequencing was summarized in Table 2 and Table 3, respectively. For detecting resistance to various drugs, the concordance between the RDBH assay and the phenotype DST varied from 77.2–95.0%, with the Kappa values ranging from 0.43–0.90, whlist the concordance between the RDBH assay and sequencing varied from 93.4–99.1%, with the Kappa values ranging from 0.85–0.98.

**Table 2:** The accuracy performance of RDBH assay compared to phenotypic DST in *Mycobacterium tuberculosis* clinical strains

Drug	Phenotypic DST	RDBH assay							
		R	S	Sensitivity (%)	Specificity (%)	PPV (%)	NPV (%)	Concordance (%)	Kappa value
RIF	R	171	14	92.4	98.5	98.8	90.5	95.0	0.90
	S	2	133						
INH	R	186	20	90.3	97.3	98.4	84.7	92.8	0.85
	S	3	111						
SM	R	120	35	77.4	91.5	90.0	81.1	84.7	0.69
	S	14	151						
EMB	R	51	32	61.4	82.7	55.4	86.0	77.2	0.43
	S	41	196						

*S* Susceptible, *R* Resistant, *PPV* Positive predictive value, *NPV* Negative predicte value, *RIF* Rifampicin, *INH* Isoniazid, *SM* Streptomycin, *EMB* Ethambutol, *RDBH* Reverse dot blot hybridization, *DST* Drug susceptibility testing

**Table 3:** The accuracy performance of RDBH assay compared to sequencing in *Mycobacterium tuberculosis* clinical strains

Drug	Sequencing	RDBH assay							
		R	S	Sensitivity (%)	Specificity (%)	PPV (%)	NPV (%)	Concordance (%)	Kappa value
RIF	M	170	4	97.7	97.9	98.3	97.3	97.8	0.96
	W	3	143						
INH	M	189	4	97.9	100.0	100.0	96.9	98.8	0.97
	W	0	127						
SM	M	134	3	97.8	100.0	100.0	98.4	99.1	0.98
	W	0	183						
EMB	M	90	19	82.6	99.1	97.8	91.7	93.4	0.85
	W	2	209						

*M* Mutated, *W* Wild type, *S* Susceptible, *R* Resistant, *PPV* Positive predictive value; NPV, Negative predicte value, *RIF* Rifampicin, *INH* Isoniazid, *SM* Streptomycin, *EMB* Ethambutol, *RDBH* Reverse dot blot hybridization

### Comparisons of the RDBH assay, phenotypic DST and sequencing for rifampicin resistance detection

Compared to the results of phenotypic DST, 171 (92.4%) out of 185 phenotypic RIF resistant strains were identified as RIF-resistant isolates by the RDBH assay (Table 2). Among 14 inconsistent isolates, 10 were not found mutations in *rpoB* by sequencing. The remaining four isolates were identified as RIF-sensitive by the RDBH assay but shown harboring mutations in *rpoB* 513, 522 or 529 by sequencing. Of 135 phenotypic RIF-susceptible isolates, 133 and 2 were identified as RIF susceptible and resistant by the RDBH assay, respectively. The two inconsistent isolates were subsequently confirmed by sequencing to have mutations at codon 511 and 531 respectively.

Sequencing results showed that 174 *M. tuberculosis* strains were found to have alterations in the RRDR of *rpoB*. The most predominant mutations in *rpoB* among these isolates were in the codons 531, 526 and 533, which were found in 82.2% (152/185) phenotypic RIF-resistant isolates totally. Of the 174 *M. tuberculosis* carried mutations in *rpoB,* 170 (97.7%) were identified as mutated and determined as RIF resistant by the RDBH assay (Table 3). Three out of 151 strains carried wild type *rpoB* confirmed by sequencing were diagnosed as mutated strains and determined as RIF resistant by the RDBH assay, two of which were found negative results in the 509–514 WT probe and identified as mutated strains, the remaining one showed mutation at codon 531.

### Comparisons of the RDBH assay, phenotypic DST and sequencing for isoniazid resistance detection

A total of 206 phenotypic INH-resistant and 114 INH-sensitive clinical isolates have been examined by the RDBH assay. One hundred and eighty-six out of 206 (90.3%) phenotypic INH resistant isolates were identified as INH resistant by the RDBH assay (Table 2). Among the 22 phenotypic INH resistant but the RDBH assay identified as INH susceptible isolates, 20 were not found mutations in the sequenced *katG*, *inhA* promoter and *oxyR-ahpC* intergenic region, two were found carrying mutations either in *katG* 315 or *inhA* (− 13) by sequencing. One hundred and eleven out of 114 phenotypic INH susceptible strains indicated positive results in all of the WT probes of *katG*, *inhA* promoter and *oxyR-ahpC* and identified as INH susceptible by the RDBH assay. For the remaining three phenotypic INH susceptible isolates that were identified as resistant by the RDBH assay, were found to harbor mutations *inhA* C(− 15) T or *katG* Ser315Thr by sequencing.

According to the sequencing results, 193 *M. tuberculosis* isolates carried mutations in *katG, inhA* promoter*,* or *oxyR-ahpC.* Of the three regions sequenced, the *katG* gene showed the highest frequency of mutations, found in 74.3% (153/206) phenotypic INH resistant isolates. Mutations in the *inhA* promoter and *ahpC-oxyR* intergenic region were only found in 26 (12.6%) and 14 (6.8%) phenotypic INH resistant *M. tuberculosis* isolates, respectively. Among 193  *M. tuberculosis* isolates that carried mutations in *katG, inhA* promoter*,* or *oxyR-ahpC* based on sequencing, 189 (97.9%) were identified as mutated and INH resistant by the RDBH assay (Table 3). None of 127 isolates carried wild types of *katG*, *inhA* promoter and *oxyR-ahpC* confirmed by sequencing were diagnosed as mutated strains and then recognized as INH susceptible by the RDBH assay.

In the present study, probes for detecting mutations in the *oxyR-ahpC* region were included. The results showed that the sensitivity of the RDBH assay compared to the phenotypic DST were increased from 83.5 to 90.3%, and increased from 90.7 to 97.9% compared to sequencing.

### Comparisons of the RDBH assay, phenotypic DST and sequencing for streptomycin resistance detection

As shown in Table 2, among 155 phenotypic SM resistant strains, 120 were identified as SM resistant by the RDBH assay. Of the 35 remaining isolates which were identified as SM susceptible by RDBH, 32 were confirmed to carry wild type of *rpsL* and *rrs* and 3 were found to carry mutations at codon 43 or 88 in *rpsL* by sequencing. Among 165 phenotypic SM susceptible strains, 151 were identified as SM susceptible by the RDBH assay. The remaining 14 isolates were detected to have alteration in *rpsL* or *rrs* gene by sequencing.

Sequencing results showed that 137 *M. tuberculosis* strains carried mutations in *rpsL* or *rrs.* Mutations *rpsL* Lys43Arg and Lys88Arg were the most prevalent mutations, found in 67.1% (104/155) of the phenotypic SM resistant isolates. Among 137 *M. tuberculosis* strains that possessed substitutions in *rpsL* or *rrs* according to the sequencing results, 134 (97.8%) were accurately determined as mutated and identified as SM resistant by the RDBH assay (Table 3). For the three inconsistent isolates, two isolates which both had mutation of *rpsL* 43 AAG-AGG and one had mutation of *rpsL* 86 CGG- CAG were considered as non-mutated by RDBH assay. Meanwhile, all of the 183 isolates that did not show any mutations based on sequencing were correctly diagnosed as non-mutated and identifies as SM susceptible by the RDBH assay.

### Comparisons of the RDBH assay, phenotypic DST and sequencing for ethambutol resistance detection

Among 83 phenotypic EMB resistant strains, 51 were identified as EMB resistant and 32 were identified as EMB susceptible by the RDBH assay (Table 2). Among the 32 inconsistent strains, 10 were found mutations in *embB* by sequencing: four carried *embB* 406 GGC-GAC (Gly-Asp), two carried 306 ATG-ATA (Met-Ile), two carried 334 TAC-CAC (Tyr-His), one carried 319 TAT-TCT (Tyr-Ser), one carried 354 GAC-AAC (Asp-Asn). Of 237 phenotypic EMB susceptible isolates, 196 were identified as EMB susceptible by the RDBH assay. The remaining 41 isolates identified as EMB resistant by the RDBH assay, 40 were found mutations in *embB*, 1 were found to have wild type of *embB*.

Sequencing results showed that 109 *M. tuberculosis* strains, including 60 phenotypic EMB resistant and 49 phenotypic EMB susceptible isolates carried mutations in *embB*. Among these 109 isolates, 90 were correctly identified as mutated by the RDBH assay (Table 3). The remaining 19 isolates which could not been identified as mutated by the RDBH assay carried mutations in *embB* found by sequencing as follows: seven carried 406 GGC-GAC (Gly-Asp), two carried 406 GGC-AGC (Gly-Ser), two carried 306 ATG-ATA (Met-Ile), one carried 306 ATG-GTG (Met-Val), two carried 334 TAC-CAC (Tyr-His), two carried 328 GAT-TAT (Asp-Tyr), one carried 319 TAT-TGT (Tyr-Cys), one carried 319 TAT-TCT (Tyr-Ser). Among 211 *M. tuberculosis* isolates found no mutations in the sequenced region of *embB*, two were identified as mutated by the RDBH assay.

### Prediction on multidrug resistance by the RDBH assay

A total of 160 phenotypic MDR isolates and 160 phenotypic non-MDR isolates were examined by the RDBH assay. One hundred and thirty-one out of 160 phenotypic MDR isolates were correctly identified as MDR by the RDBH assay. According to the sequening results, 141 isolates carried mutations both in RIF resistant associated gene *rpoB* and INH resistant associated genes *katG*, *inhA* promoter or *oxyR-ahpC* interegenic, and were recognized as genotypic MDR. Among the 141 genotypic MDR isolates, 130 were correctly identified as MDR by the RDBH assay. None of phenotypic non-MDR and one genotypic non-MDR isolate was misclassified as MDR by the RDBH assay.

## Discussion

The emergence and spread of MDR tuberculosis and XDR tuberculosis pose a serious impediment to global tuberculosis control. Early selection of appropriate treatment is vital to achieve good prognosis for the patients. Currently, molecular diagnostic methods, based upon the identification of specific gene mutations associated with drug resistance, are the most promising techniques for rapid detection of drug resistant *M. tuberculosis* clinical strains. Recently, some commercial assays, such as GenoType MTBDR*plus,* GenoType MTBDR*sl,* FluoroType MTBDR (Hain Lifescience GmbH, Nehren, Germany), GeneXpert MTB/RIF, Xpert MTB/RIF Ultra Assay (Cepheid Corp., USA), started to be used in many countries [11–14, 19–21]. These methods have sensitivities ranging from 85.56 to 100% and specificities spanning 78.26–100% for RIF resistance, and sensitivities from 61.6 to 100% and specificities spanning 66.7–100% for INH resistance by testing culture isolates of *M. tuberculosis* compared with a culture-based DST reference standard (11, 21, 22). While GeneXpert MTB/RIF and Xpert MTB/RIF Ultra is limited to the detection of RIF resistance [22], GenoType MTBDR*plus* is able to identify both INH and RIF resistance [11]. In this study, a RDBH assay was established to simultaneously diagnose RIF, INH, SM and EMB resistance. It takes only 7 h for identification after extraction DNA from L-J cultures and allows the simultaneous analysis of 42 clinical DNA samples for four drug resistance detection. In the present study, we combined the susceptibility results of RIF and INH from the RDBH assay, and found that 82% phenotypic MDR isolates and 92% genotypic MDR isolates been correctly identified. The ability of the RDBH assay for predicting multidrug resistance is directly affected by its ability for predicting INH and RIF resistance.

As for RIF, four WT probes and seven MT probes were designed to target mutations in RRDR, 92.4% (171/185) phenotypic RIF-resistant strains and 97.7% (170/174) strains carrying mutations in *rpoB* were successfully determined by this analysis. We attributed the high sensitivity mainly to the wide coverage of diverse of mutation types. Compared with the principle of GenoType MTBDR*plus*, our RDBH assay excluded MT probes targeting codon 505–509 mutation because of their low frequencies of occurrence [12], but added four additional MT probes that were able to detect common mutations of Leu511Pro, Asp516Gly, Asp516Tyr, and His526Arg. Previous studies from different geographical locations have evaluated the use of RDBH assay in detecting RIF resistance and reported the sensitivity ranging from 85.6 to 100% [15, 23, 24]. In our set of strains, codon 531, 526 and 533 were the most frequently detected sites of *rpoB* mutations associated with RIF resistance, accounting for 45.9, 29.1, and 8.6% respectively, which were contrasted to many other reports shown that the three most prevalent mutation loci in *rpoB* were 531, 526 and 516 [7, 25]. In addition, mutations were not observed in 14 RIF-resistant isolates, suggesting that there may be mutations outside the RRDR or elsewhere in the genome. Alternatively, the resistance might be due to the other underlying mechanisms, such as drug efflux pump or decreased permeability of the outer membrane [26]. Moreover, seven double-loci mutations were detected successfully by our assay, indicating that our method also had good sensitivity in detecting multiple loci mutations.

Our results showed that the use of RDBH assay could detect 73.3% (151/206) of phenotypic INH resistant isolates due to the presence of *katG*315 mutation. This could be further increased to 90.3% (186/206) if both the *inhA* promoter and *oxyR-ahpC* intergenic region mutations were involved. The sensitivity of this study was comparable to that reported from Pakistan (90.6%) [27] but much higher than that from China (80.25%) [28] which both used GenoType MTBDR*plus.* We speculated that the difference between this study and another study from China [28] may be attributed to that the mutation prevalence of *oxyR-ahpC* intergenic region were showed in 5–20% INH resistant *M. tuberculosis* in China [5, 29, 30], however, GenoType MTBDR*plus* did not include probes targeted in *oxyR-ahpC* intergenic region. Previous studies showed that mutations in *oxyR-ahpC* intergenic region could compensate for loss of KatG/CP activity caused by mutations in *katG* [31] or were directly associated with low level INH resistance [32]. In our study, a 6.8% (14/206) increase in the sensitivity was obtained by involving probes for the *oxyR-ahpC*. However, our method failed to detect four INH-resistant isolates, which harbored mutations in *katG* Ala312Glu, *katG* Trp191Gly, *inhA* G(− 13) T and *ahpC* G(− 48) A, respectively. *katG*191 and *katG*312 were not included in the coverage of our targeted region due to the poor relevant to INH resistance, and isolates harbored mutations in *inhA* G(− 13) T and *ahpC* G(− 48) A showed ambiguous signals in the corresponding WT loci so that might be misjudged by readers of the RDBH assay. So, improvement of the specificity of the WT probes will be needed to avoid false negative results.

Farhat et al. reported that the *embB* M306I and M306V mutations were significantly associated with INH resistance even after stratification by the EMB resistance status [7]. At the present study, we found that statistics significance were found between *embB* mutations and INH resistance among the EMB susceptible isolates (*χ*^2^ = 23.76, *P* = 0.000) but not among EMB resistant isolates (*χ*^2^ = 0.875, *P* = 0.349). Since previous studies have showed that *embB* M306I and M306V mutations were associated with INH resistance [7, 33], we added the results according to the probes targeted at *embB306* and found that the sensitivity increased from 90.3% (186/206) to 94.2% (194/206) whilst the specificity dropped from 97.3% (111/114) to 92.1% (105/114) compared to the phenotypic DST, so we suggested that *embB306* mutation had limited effect on predicting INH resistance though there may be association between *embB* mutations and INH resistance.

Regarding SM, RDBH assay was able to detect 120/155 SM phenotypic resistant samples (sensitivity 77.4%) and 91.5% susceptible isolates by detecting mutations in *rpsL* and *rrs* genes. There were 14 samples found to contain mutations by both the RDBH assay and sequencing but were susceptible to SM based on phenotypic DST results. Phenotypic DST assay (L-J based proportional method) was repeated for these samples to confirm the results. Similar phenomenon was also observed by Zhang et al. [34], which could be attributed to the low-level SM-resistance that showed false-negative results by phenotypic DST. Regarding the 35 SM phenotypic resistant samples were detected as wild type by the RDBH assay, additional mechanisms which have been shown to be associated with SM resistance might be involved, e.g., cell membrane permeability changes [35], alterations in other genes, such as the *gidB* gene [36]. The mutations in *rpsL* were known to relate to high-level SM resistance whereas *rrs* mutations were mainly related with low-level or intermediate-level SM resistance, identification of mutation types offered more information to understand the relationship between the phenotypic and genotypic feature of SM-resistant *M. tuberculosis* [37]. When compared with sequencing, the sensitivity and specificity of RDBH assay were 97.8% (134/137) and 100.0% (183/183), respectively. Three isolates were diagnosed as unmutated by RDBH. Of these two harbored *rpsL* Lys43Arg mutation and the remaining one had *rpsL* Arg86Gln alteration. This suggests that further optimizing experimental conditions would improve the sensitivity.

As reported elsewhere, substitutions in codon *embB*306 are the predominant mechanism conferring EMB resistance, which accounted for 48.3–70.6% resistant isolates [38–41]. Among EMB-resistant isolates, we found that 72.3% (60/83) were observed to carry *embB* mutation and 56.6% (47/83) carried mutations in codon 306 of *embB*, which was consistent with the previous investigations [38–41]. So, we speculated that mutations in *embB* was associated with EMB resistance*.* Using the RDBH assay, we obtained a moderate sensitivity (61.4%) and specificity (85.7%) compared with phenotypic DST and a higher sensitivity (82.6%) and an excellent specificity (99.1%) compared with sequencing. A multicenter evaluation by Mokrousov et al. [42] found lower sensitivity (51.0%) and specificity (82.6%) by only covering codon 306. The improved detection efficiency might be attributed to that our assay covered three more probes targeted *embB*406. However, we also found that nine isolates carried mutation at *embB*406 were not identified by the RDBH assay, so the experimental conditions like reagent proportions, reaction temperatures should be further improved, or designing new probes targeted *embB*406 was needed. The discrepancy between the phenotypic DST and RDBH assay could be due to either a poor performance of the conventional DST method [43–45]. Clarifying the resistance mechanisms of EMB, such as *embA*, *embC* and *embB* codon 497, may also improve the performance of the molecular DST for predicting EMB resistance.

## Conclusions

The multiplex PCRs based RDBH built in our study could determine three first-line drugs and one second-line drug resistance of 1 to 42 *M. tuberculosis* samples within 7 h, and showed high consistency to phenotypic DST method and sequencing, suggesting that it is an outstanding diagnostic tool for simultaneously determining the resistance to RIF, INH, SM and EMB and is especially suitable for application in tuberculosis laboratories with heavy testing tasks.

## Supplementary information

**Additional file 1: Supplemental Table 1** Interpretations of the RDBH assay, the phenotypic resistance and sequencing results for 41 *Mycobacterium tuberculosis* in Fig. 1.

## Data Availability

The datasets used and/or analysed during the current study are available from the corresponding author on reasonable request.

## Abbreviation

AP-NBT/BCIP: Alkaline phosphatase–nitroblue tetrazolium chloride/5-bromo-4-chloro-3-indolyl pholsphate
DST: Drug susceptibility testing
EMB: Ethambutol
INH: Isoniazid
L-J: Lowenstein-Jensen
MDR: Multidrug-resistant
MT: Mutant
NPV: Negative predictive value
PLAD: Provincial-level administration division
PPV: Positive predictive value
RDBH: Reverse dot blot hybridization
RIF: Rifampicin
RRDR: Rifampicin resistance-determining region
SDS: Sodium dodecyl sulfate
SM: Streptomycin
SSPE: Saline–sodium phosphate–ethylenediaminetetraacetic acid
TMB: 3, 3′, 5, 5′-Tetramethylbenzidine
WHO: World Health Organization
WT: Wild type
XDR: Extensively drug-resistant
